# Diurnal Variations of Depression-Related Health Information Seeking: Case Study in Finland Using Google Trends Data

**DOI:** 10.2196/mental.9152

**Published:** 2018-05-23

**Authors:** Jonas Christoffer Tana, Jyrki Kettunen, Emil Eirola, Heikki Paakkonen

**Affiliations:** ^1^ Department of Health and Welfare Arcada University of Applied Sciences Helsinki Finland; ^2^ Information Studies School of Business and Economics Åbo Akademi University Turku Finland; ^3^ Department of Business Management and Analytics Arcada University of Applied Sciences Helsinki Finland

**Keywords:** depression, consumer health information, information seeking behavior, infoveillance, infodemiology, mental health, search engine

## Abstract

**Background:**

Some of the temporal variations and clock-like rhythms that govern several different health-related behaviors can be traced in near real-time with the help of search engine data. This is especially useful when studying phenomena where little or no traditional data exist. One specific area where traditional data are incomplete is the study of diurnal mood variations, or daily changes in individuals’ overall mood state in relation to depression-like symptoms.

**Objective:**

The objective of this exploratory study was to analyze diurnal variations for interest in depression on the Web to discover hourly patterns of depression interest and help seeking.

**Methods:**

Hourly query volume data for 6 depression-related queries in Finland were downloaded from Google Trends in March 2017. A continuous wavelet transform (CWT) was applied to the hourly data to focus on the diurnal variation. Longer term trends and noise were also eliminated from the data to extract the diurnal variation for each query term. An analysis of variance was conducted to determine the statistical differences between the distributions of each hour. Data were also trichotomized and analyzed in 3 time blocks to make comparisons between different time periods during the day.

**Results:**

Search volumes for all depression-related query terms showed a unimodal regular pattern during the 24 hours of the day. All queries feature clear peaks during the nighttime hours around 11 PM to 4 AM and troughs between 5 AM and 10 PM. In the means of the CWT-reconstructed data, the differences in nighttime and daytime interest are evident, with a difference of 37.3 percentage points (pp) for the term “Depression,” 33.5 pp for “Masennustesti,” 30.6 pp for “Masennus,” 12.8 pp for “Depression test,” 12.0 pp for “Masennus testi,” and 11.8 pp for “Masennus oireet.” The trichotomization showed peaks in the first time block (00.00 AM-7.59 AM) for all 6 terms. The search volumes then decreased significantly during the second time block (8.00 AM-3.59 PM) for the terms “Masennus oireet” (*P*<.001), “Masennus” (*P*=.001), “Depression” (*P*=.005), and “Depression test” (*P*=.004). Higher search volumes for the terms “Masennus” (*P*=.14), “Masennustesti” (*P*=.07), and “Depression test” (*P*=.10) were present between the second and third time blocks.

**Conclusions:**

Help seeking for depression has clear diurnal patterns, with significant rise in depression-related query volumes toward the evening and night. Thus, search engine query data support the notion of the evening-worse pattern in diurnal mood variation. Information on the timely nature of depression-related interest on an hourly level could improve the chances for early intervention, which is beneficial for positive health outcomes.

## Introduction

### Background

Temporal variations and clock-like rhythms govern many different health-related behaviors and complications [1,2]. Partly because of the methodological barriers presented by traditional means of collecting data, research has mainly focused on longer temporal scale patterns, such as annual or seasonal variations, rather than shorter scale patterns such as weekly or hourly rhythms [2]. However, recent advances in information and communication technology are emerging to fill some of these methodological gaps in public health surveillance research [2]. Most health-related behaviors on the internet, such as health information seeking, leave digital footprints that can be traced with surprising accuracy. Having a broader understanding of these temporal variations can have great public health advantages. Internet health information seeking related to different health problems is common everyday behavior, and daily, millions of people seek help on the internet, ranging from identifying symptoms to finding diagnoses and treatments [3-5]. In Finland, internet use is ubiquitous, also in relation to health, as 66% of the Finnish population aged 16-89 years search for information related to health. For the younger demographic segment aged 25-34 years, the percentage is 82 [6]. More generally, it has been estimated that between 70% and 90% of health care is undertaken by individuals without the involvement of health care professionals [7,8]. This would suggest that before seeking treatment from health care professionals, people will first try to treat their problems themselves, with search engines as the most common approach [7-11]. The most popular search engine in the world, Google [12], had a market share of 96% in Finland in 2016 [13]. This kind of information behavior reveals the information seekers’ thoughts and actions in relation to interests, causes, symptoms, advices, and cures that people who suffer from poor health or illness develop [1,14-16]. As said, it also leaves digital footprints, or user-generated online data, which are easily accessible in near real-time, and allows for identifying and proposing new hypothesis as well as open up unique opportunities to investigate health-related behaviors on a gigantic scale [17-19].

### Objective

Several studies, ranging from seasonal influenzas [20-23], viruses [24], and general diseases surveillance [25-30] to mental health problems [1,2,17,31,32], have demonstrated that valid trends and insights reflecting entire populations have been extracted from online data, particularly search engine data. Predicting the incidence of both communicable and noncommunicable diseases has been shown reliable [15,18,23,33]. This utilization of online data for health care research is especially useful when studying phenomena where little or no traditional data exist, and has even led to an emerging field of research called infodemiology, defined by Eysenbach [9] as the science of the distribution and determinants of information in an electronic medium, specifically the internet, or in a population, with the ultimate aim to inform public health and public policy [9,15,18,34]. One specific area where traditional data are incomplete is the study of diurnal variations of depression. In depression-related health information–seeking behavior, seasonal variations have been identified with the help of search engine data [17,31]. Thus, one possible way of studying diurnal variations of mood is analyzing information-seeking behavior with the help of search engine data, which are available on an hourly level. To the authors’ best knowledge, no study has investigated how search engine variables perform as predictors for interest in depression on an hourly level. Therefore, the aim of this exploratory study was to analyze diurnal variations for interest in depression on the Web to discover hourly patterns of depression interest and help seeking.

Depression is one of the most burdensome diseases in the world [35]. Each year about 7% of the global population suffers from major depression and 25% suffers from anxiety or milder forms of depression [35]. In Finland, at least 5% of the adult population suffers from depression every year [36]. One of the characteristics of depressive mood is diurnal mood variation (DMV). DMV refers to noticeable diurnal or daily changes in the overall mood state experienced by individuals suffering from depression as well as healthy individuals [37-39]. The occurrence of diurnal variations has been shown to be somewhat irregular, and the presence and direction of mood variations are decidedly inconclusive over time, with evidence for both morning-worse and evening-worse peaks in emotional distress [37,38,40]. There are very few studies recording DMV at distinct periods of time, partly due to the limitations of ways to monitor DMV [37]. As individuals suffering from mental health issues have been shown to be more likely to seek information about their problems on the internet, studying search engine data and health information–seeking behavior has the potential to provide new meaningful insights into DMVs [8,31,37,41].

## Methods

### Study Design

Search data generated in Google can be accessed via Google Trends, a public Web-based database that provides time series data of search trends. Data from Google Trends are provided as relative search volumes (RSVs), which, in contrast to raw or absolute search volumes, is corrected and adjusted over time due to changes in internet access or disposable time (for example, all searches may decline during Saturday) [1,42]. correct changes in search volume variations due to availability of internet access or disposable time [1,42]. Queries in Google Trends are monitored relative to all queries, in this case each hour, and reported as RSV, where RSV=100 is the hour with the highest search proportion for the day, and RSV=50 is 50% of that highest proportion. Queries from search activity on Google are disaggregated to geographical and temporal units, ranging from years to minutes, and deidentified from any identifying information to protect user privacy. In this study, the following 6 Finnish search queries related to depression were monitored “Masennus” (Eng. depression), “Masennus oireet” (Eng. depression symptoms), “Masennustesti” (Eng. depression test), “Masennus testi” (Eng. depression test), “Depression,” and “Depression test” (the Swedish as well as the English term for depression and depression test). Query terms were not used in combination and not with quotes around the search terms. Additional terms to the root term depression were added using Google Trends–related terms function, which identifies associated top search terms by either content or users’ search behavior [42]. The query terms were chosen to represent an extensive sample of queries related to depression, in general, as well as symptoms and self-diagnosis, in Finnish and Swedish, the 2 official languages of Finland. The search queries were specified to Finland as a geographic area to avoid mixing with search queries originating elsewhere. Data were downloaded on a weekly basis from Google Trends throughout March 2017. March was chosen as the prevalence of depression in Finland has been shown to peak in spring [43]. Weekly downloads enabled obtaining hourly search volume data for each day in March, resulting in a RSV value for 744 hours for the 6 different search terms (n=4464), ranging from March 1 to 31, 2017. The analysis in this study are based on public meta-data that do provide neither information about the race, gender, age, or any other identifying information of the person entering a search term nor involve any intervention in the integrity of a person. Therefore, no institutional board review was required.

### Wavelet Power Spectrum Analysis

**Figure 1:**
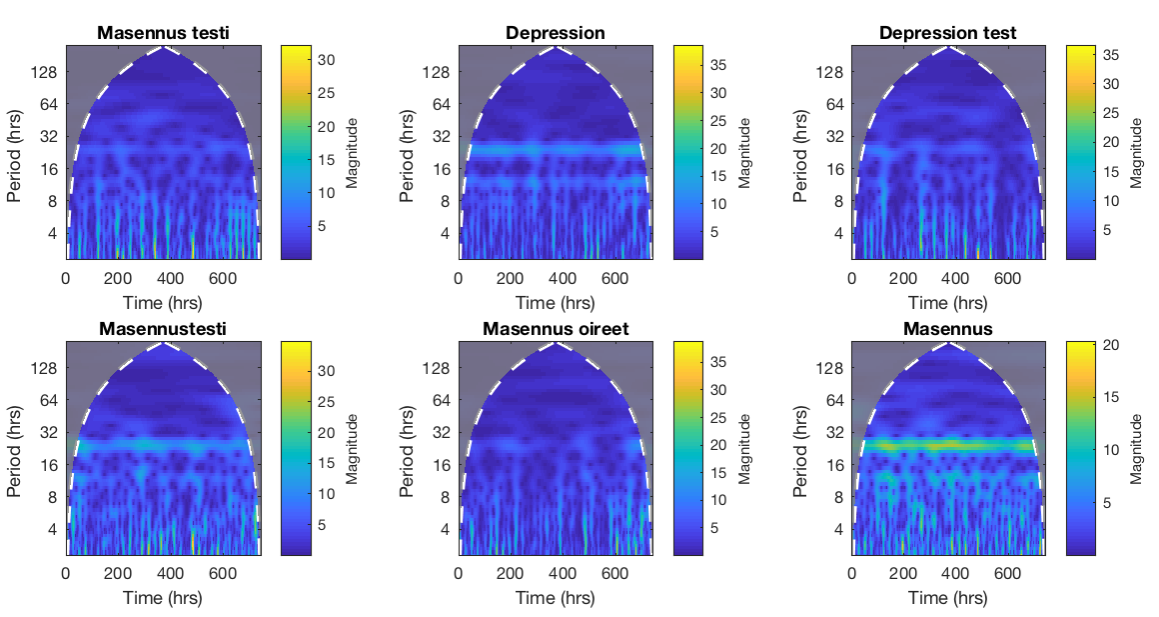
Wavelet power spectra for the relative search volumes (RSVs) of each query.

**Figure 2:**
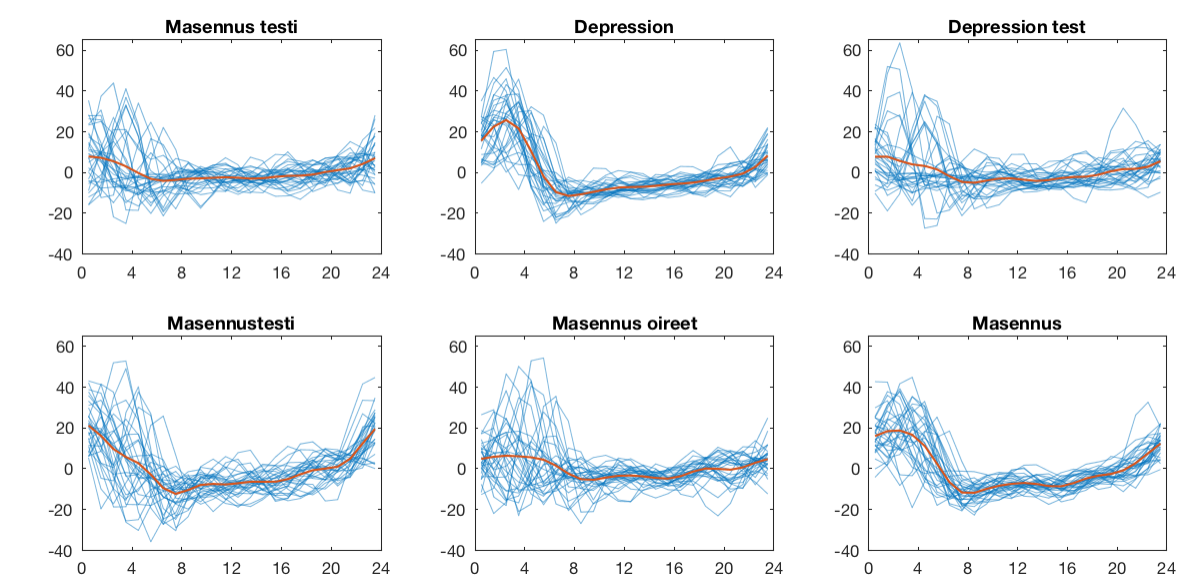
Each 24-hour period (in blue) of the reconstructed signal superimposed with its arithmetic mean (red), separately for each query.

**Figure 3:**
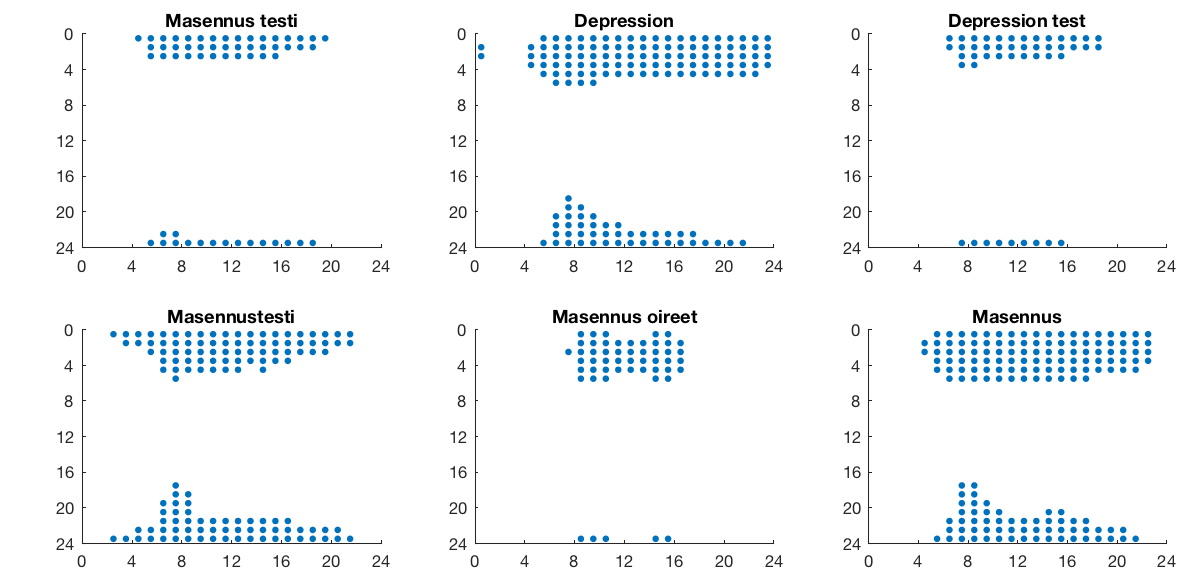
Plots for each query indicating between which hours of day the differences are statistically significant. Each marked dot means that the interest for the hour on the y-axis is significantly greater than that for the hour on the x-axis.

A continuous wavelet transform (CWT) was applied to the hourly data to focus on the diurnal variations. The CWT and subsequent analysis were conducted with MathWorks Matlab R2017b. The wavelet power spectra (WPS) for the RSVs of each query are shown in Figure 1. To extract the diurnal variation, longer term trends were removed by discarding components with a period of longer than 32 hours. Similarly, components with a period of less than 4 hours were considered noise, and also eliminated. Calculating the inverse transform of the remaining part of the CWT thus provides a reconstruction of the signal without the trends and noise. The reconstructed data are presented in Figure 2, with all the days superimposed with their arithmetic mean, separately for each query. An analysis of variance was conducted to determine the statistical differences between the distributions of each hour, applying the Tukey-Kramer procedure for multiple comparisons. The results are summarized in Figure 3, where each marker represents a statistically significant (alpha=.05) difference between the interests of the corresponding hours.

### Trichotomization

The hourly data were also trichotomized and analyzed in 3 time blocks: 8.00 AM to 3.59 PM, 4.00 PM to 11.59 PM, and 0.00 AM to 7.59 AM. The trichotomization of the 24 hours of a day makes it possible to distinguish and compare structured time (office hours), unstructured time (leisure time) as well as nighttime. All hourly RSV values between 0 and 100 for each search term were added to the set time blocks. Differences between search volumes in the 3 time blocks were then calculated. Analysis of variance was conducted to calculate statistical differences in the distributions between the different time blocks. In post hoc between-group (time blocks) comparisons, the Bonferroni correction was used. *P* ≤.05 was considered statistically significant. The trichotomization analyses for were performed with SPSS (version 24.0; SPSS Inc., Chicago, Illinois, USA).

## Results

### Wavelet Power Spectrum Analysis

The main results of this study are shown in Figures 1-3 and Multimedia Appendix 1. The daily variability is identifiable in each figure of the WPS (Figure 1) as a consistent signal with elevated power for components with a period around 24 hours, and is most distinctly observable in the case of “Masennus.”

As can be seen in Figure 2, the search volumes for all depression-related query terms showed a unimodal regular pattern during the 24 hours of the day for the time period studied. All queries feature clear peaks during the nighttime hours around 11 PM to 4 AM and troughs between 5 AM and 10 PM.

The analysis of variance (Figure 3) reveals that search interest during nighttime hours 11 PM to 3 AM is greater than the interest during daytime hours 8 AM to 4 PM, with some variation for different queries.

In the means of the CWT-reconstructed data, the differences in nighttime and daytime interest are evident. As the original data were scaled to 100 for the maximum weekly RSV, the units here are in percentage points (pp) of that value. The largest difference between peaks and troughs is visible for the query term “Depression,” with a difference of 37.3 pp (max. at 2-3, min. at 7-8), followed by “Masennustesti,” with a difference of 33.5 pp (max. at 0-1, min. at 7-8), and “Masennus,” with a difference of 30.6 pp (max. at 2-3, min. at 8-9). “Depression test” (difference 12.8 pp, max. at 0-1, min. at 8-9), “Masennus testi” (difference 12.0 pp, max. at 0-1, min. at 6-7), and “Masennus oireet” (difference 11.8 pp, max. at 2-3, min. at 9-10) showed smaller differences, with fewer statistically significant differences during the 24-hour period.

### Trichotomization

Analysis of mean search activity of the 3 time blocks showed peaks in the first time block (00.00 AM-7.59 AM) for all 6 terms. The mean search volumes then decreased significantly during the second time block (8.00 AM-3.59 PM) for the terms “Masennus oireet” (*P*<.001), “Masennus” (*P*=.001), “Depression” (*P*=.005), and “Depression test” (*P*=.004). Thereafter, the mean search volume between the second and the third time block (4.00 PM-11.59 PM) rose again for the term “Masennus oireet” (*P*=.04). Furthermore, there was a tendency for higher mean search volumes for the terms “Masennus” (*P*=.14), “Masennustesti” (*P*=.07), and “Depression test” (*P*=.10) between the second and third time blocks. Multimedia Appendix 1 shows the search volumes and standard deviations for various search terms in the 3 time blocks.

## Discussion

### Principal Findings

This study provides novel, rapid, cost-effective, and efficient internet-based evidence for insights in to the diurnal variations of depression-related health information seeking. The results of this study suggest that help seeking for depression has clear diurnal patterns, with significant rise in depression-related query volumes toward the evening and night. Our findings support the notion of the previously identified evening-worse DMVs, which is thought to be associated with milder depressive symptoms [40]. The nighttime peaks in search volumes also support the notion of poor sleep quality in subjects with depression and the causal relationship between insomnia and depression noted by Tsuno et al [44]. One interpretation of this finding, supported by Rusting and Larsen [40], is that structured versus unstructured time may account for DMVs. Evening time, for most people, is less constrained by external demands and obligations, and the most likely time of day in which negative thinking may take place, compared with the more structured working hours during the day. According to Hasler [37], the specific patterns of these diurnal mood changes are assumed to characterize different subtypes of depression. DMV has been linked most closely to melancholic depression, characterized by a pattern of feeling worst in the morning, and to atypical depression, which is characterized by the evening-worse pattern [37]. Among both depressed and nondepressed individuals, the evening-worse pattern is thought to be associated with milder depressive symptoms, and may characterize chronic dysthymia with neurotic-type symptoms of depression, including responsivity of mood, initial insomnia, self-pity, hypochondriasis, hopelessness, and anxiety [40,45,46]. On the basis of the results in this study, more Finnish people in need of help may require support during nighttime rather than during office hours. In addition, as the evening-worse pattern is associated with milder depressive symptoms, early intervention could be beneficial for positive health outcomes. Traditional epidemiological surveys on DMVs and depression suffer from data-related issues, such as long-term data retrieval, collection, and processing. Earlier research on DMVs has been based on self-ratings of mood, using visual analogue scales and retrospective assessment [38], where patients have been asked to describe the pattern retroactively, either during a clinical interview or in response to an item in a questionnaire [37]. In this respect, infodemiology, and especially search engine data, which is a relatively new concept, presents a promising complementary approach to population and public health research. As stated by Nuti et al [33], Google Trends has potential to afford meaningful insights into population behavior and its link to health and health care. The advantages of monitoring and mining search engine data also lie in the cost-effective and near real-time analysis it enables. In this case, gathering data at hourly intervals using conventional techniques such as surveys would be expensive, time–consuming, and difficult. The strength of this type of big data analysis is that it allows to draw conclusions based on populations of information units rather than on an individual level [34].

### Limitations

It needs to be noted that the results of this study are based on query terms, and there is a challenge in interpreting the semantics of Google queries. It is not clear why a person is searching for the keyword in question, and individual search queries do not necessarily reflect the actual mood state of the user [17,34]. Therefore, it is important to note that the variables used in this paper present interest and not actual incidence. However, as earlier research using search query analysis has shown, it is reasonable to assume that the reason people seek health information about a specific symptom on the internet is because they, or people they know, may be experiencing the symptoms in question [17]. These diurnal trends of help seeking can also provide information that may be useful in further hypothesis testing. As already stated, the internet is by far the most popular vehicle for health information seeking, and changes in health status are often reflected in immediate changes in information and communication patterns on the internet [15,34]. Today, people are more inclined to self-diagnose and seek information to improve their understanding of their personal health using information that is available online [7-9,14]. Search query volumes do not offer demographic data, thus limiting our ability to draw conclusions about population behavior in general. However, as statistics in Finland show, almost all age-by-demographic population categories seek health information on the internet, suggesting that search engine trends may reflect trends in the health of populations. The use of search engine data has, moreover, been proposed to foster and encourage infodemiological research, not to replace or substitute the need for traditional epidemiological research. Therefore, this approach should be seen as complementary to traditional surveillance methods. There are also limitations in the Google Trends database, as there are insufficiencies in detailed information on the method by which Google Trends generates search data and the specific algorithms it employs to analyze it [33].

### Implications

The findings in this study could be utilized by public health officials to facilitate aid and optimize positive health outcomes by providing resources at the best time for intervention, that is, when the majority of people with information needs related to depression are engaged in the process of information seeking. This is a time when the subjects are focusing their attention on the health threat and direct their efforts to becoming more engaged and providing ways of managing depression-related contemplations. This could increase the chances of early intervention for people in need of help or those feeling depressed or worrying about depression-related issues. As the evening-worse pattern for DMV has been linked to milder symptoms in depressed as well as nondepressed individuals [37,40,45], optimal intervention time is very important to guide individuals to optimal sources before their symptoms get worse. In addition, DMV is believed to have clinical relevance as a predictor of treatment response [38]; therefore, it is essential to have an extensive understanding of this phenomenon to help health care professionals and others to plan optimal treatments. Thus, these findings should be concomitant with strategies to ensure that those in need of help have a Web-based pathway to evidence-based information and aid, such as the Finnish mental health hub [47].

### Conclusions

This paper is a novel attempt at utilizing search engine data on an hourly level to monitor diurnal variation in interest in depression. These initial steps could be developed further in subsequent studies, with the aim to discover larger temporal variations and patterns of health contemplations on an hourly timescale. The method of studying hourly variations and trends in search engine query volumes could also be utilized in other health-related contexts, besides depression. The aim of this study was to draw attention to the possibility of gaining hourly insights into diurnal variations of depression-related information seeking by using search engine data. It is of vital importance to have a wide understanding of depression-related behavior to be able to provide the best possible treatments at the best possible time. Monitoring hourly search volume could serve as an efficient surveillance method for investigating the timely nature of depression-related help seeking. Despite its limitations, this preliminary analysis showed that monitoring hourly search volumes for depression is informative, and that the benefits of this kind of an approach are various.

## Appendix

Multimedia Appendix 1 Analysis of mean search activity of the 3 time blocks.

## Abbreviations

CWT: continuous wavelet transform
DMV: diurnal mood variation
RSV: relative search volume
WPS: wavelet power spectra
