# Supplementary figures and images for: Diurnal Variations of Depression-Related Health Information Seeking: Case Study in Finland Using Google Trends Data
Source: JMIR Ment Health. 2018 May 23;5(2):e43. doi: 10.2196/mental.9152 (PMC5990858; doi:10.2196/mental.9152)

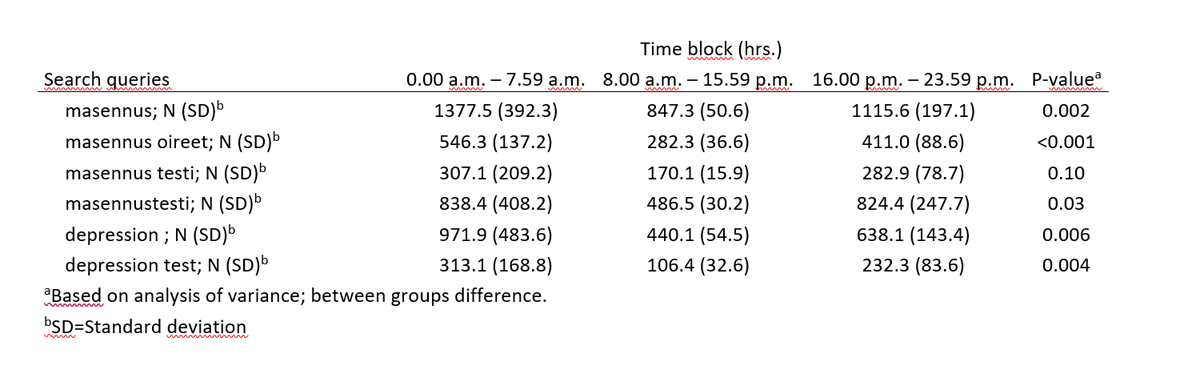

Supplement: Multimedia Appendix 1 [file mental_v5i2e43_app1.png]
